# Optimisation of Storage and Transportation Conditions of Cultured Corneal Endothelial Cells for Cell Replacement Therapy

**DOI:** 10.1038/s41598-020-58700-5

**Published:** 2020-02-03

**Authors:** Stephen Wahlig, Gary S. L. Peh, Khadijah Adnan, Heng-Pei Ang, Chan N. Lwin, F. Morales-Wong, Hon Shing Ong, Matthew Lovatt, Jodhbir S. Mehta

**Affiliations:** 1 Duke University School of Medicine, Durham, NC USA; 2 Tissue Engineering and Stem Cell Group, Singapore Eye Research Institute, Singapore, Singapore; 3 Duke-NUS Graduate Medical School, Singapore, Singapore; 4 Singapore National Eye Centre, Singapore, Singapore; 5 Autonomous University of Nuevo Leon (UANL), University Hospital, Monterrey, Mexico; 6 School of Material Science and Engineering, Nanyang Technological University, Singapore, Singapore

**Keywords:** Translational research, Eye diseases

## Abstract

As the cornea is one of the most transplanted tissues in the body it has placed a burden on the provision of corneas from cadaveric donors. Corneal endothelial dysfunction is the leading indication for cornea transplant. Therefore, tissue engineering is emerging as an alternative approach to overcome the global shortage of transplant-grade corneas. The propagation and expansion of corneal endothelial cells has been widely reported. However, one obstacle to overcome is the transport and storage of corneal endothelial cells. In this study we investigated whether tissue engineered corneal endothelial cells can be preserved in hypothermic conditions. Human corneal endothelial cells (HCEnCs) were exposed to various temperatures (4 °C, 23 °C, and 37 °C) in both adherent and suspension storage models. Optimal storage media and storage duration was tested along with post-storage viability. Following storage and subsequent recovery at 37 °C, cell phenotype was assessed by immunofluorescence, gene and protein expression, and proliferative capacity analysis. Functionality was also assessed within a rabbit model of bullous keratopathy. Our data support our hypothesis that functional HCEnCs can be preserved in hypothermic conditions.

## Introduction

The cornea is a transparent tissue composed of five layers – epithelium, Bowman’s layer, stroma, Descemet’s membrane (DM), and endothelium - which functions as the primary refractive component of the eye. The inner endothelial layer exists as a monolayer of polygonal corneal endothelial cells (CEnCs) which regulate stromal hydration through a combination of passive leakage and an active ionic pump function^1,2^. Human corneal endothelial cells (HCEnCs) are non-proliferative *in vivo*. Moreover, cell density gradually decreases over an individual’s lifetime, at a rate of approximately 0.6% per year^3,4^. Generally, cell loss can be compensated for by enlargement of adjacent HCEnCs, which maintains appropriate functional integrity essential for corneal hydration^5^. However, endothelial pathologies such as Fuchs endothelial dystrophy (FED) or pseudophakic bullous keratopathy decrease HCEnC density below a critical threshold (500–1000 cells/mm^2^), which results in dysregulation of fluid movement, corneal edema and eventually corneal blindness^6–9^.

Over 12 million people globally are affected by corneal blindness^10^. Though treatable through corneal transplantation, a lack of donor tissue availability limits this approach^5^. Surgical techniques like Descemet’s stripping automated endothelial keratoplasty (DSAEK) and Descemet’s membrane endothelial keratoplasty (DMEK), have made it is possible to use corneal layers for the treatment of multiple patients from a single donor cornea^11,12^. However, minimizing the amount of utilized tissue through procedures such as hemi-DMEK or Quarter-DMEK is insufficient^12–15^, as current estimates suggest that only 1 in 70 patients are receiving a corneal transplant^10^.

An alternative to cadaveric transplantation is cell therapy using HCEnCs propagated *in vitro*. Instead of transplanting donor tissue directly into a small number of patients, isolated endothelial cells from a single donor can be propagated to treat up to 80 patients^16,17^. Although HCEnCs are non-proliferative *in vivo*, proliferative capacity been successfully established *in vitro*^18^. Furthermore, subsequent advances in culture protocols have significantly improved corneal endothelial cell yields^17,19–22^. These expanded HCEnCs can be therapeutically administered through direct cell injection into the anterior chamber^23–26^ or implantation of biological^17,27–29^ or synthetic^30–33^ scaffolds seeded with the propagated HCEnCs. The first-in-man clinical trial of injected HCEnCs for bullous keratopathy has demonstrated encouraging results for this emerging therapy^26^.

Practical worldwide implementation of a cell therapy approach necessitates standardized protocols for storage and transportation of HCEnCs. Storage at 37 °C is currently used for maintenance of HCEnCs in the laboratory and is a popular form of donor cornea storage in Europe^34^. However, the requirement for large sterile incubators for transportation makes this approach impractical. In the USA, donor corneas are typically stored in Optisol at 2–8 °C^35,36^. However, *in vitro* cultures of HCEnCs show poor viability when stored in Optisol at 4 °C^37,38^. This likely reflects the phenotypic differences between *ex vivo* and cultures of HCEnCs, consistent with previously analysis of transcriptome data^39^. Studies in porcine CEnCs have demonstrated that cells incubated at 4 °C assume a rounded retracted morphology, that may be due to elevated oxidative stress^40^. Despite the practical benefits, cold storage for HCEnCs has not been fully explored. However, the development of specialized storage media and additives designed to mitigate oxidative stress and cold-induced injury have enabled hypothermic storage for a variety of cell types including hepatocytes^41,42^, chondrocytes^43^, and adipose-derived stem cells^44^. Most recently, Bartakova *et al*. described a method of transporting HCEnCs with preserved viability^45^. Moreover, Parekh *et al*. were able to ship DSAEK samples internationally at ambient temperatures prior to human transplantation^46^. While these reports highlight the potential for hypothermic storage of HCEnCs, the phenotype and functionality of these post-storage cells requires focused investigation to ensure they are appropriate for *in vivo* therapy.

In this study we evaluated storage media and defined optimal protocols for both 4 °C and 23 °C storage of HCEnCs. Since cell injection and HCEnC-seeded scaffolds may become viable therapeutic options in the future^47^, we tested both suspension and adherent storage models to accommodate both paradigms. In addition, we evaluated cell viability and morphology during storage. Furthermore, to assess whether stored HCEnC retain a corneal endothelial phenotype we investigated proliferative capacity, cell-surface marker, gene and protein expression of HCEnCs post-storage. Finally, functional capacity of was assessed in a rabbit model of bullous keratopathy by means of corneal endothelial cell injection (CE-CI).

## Results

### Hypothermic storage protocol optimization

**Figure 1:**
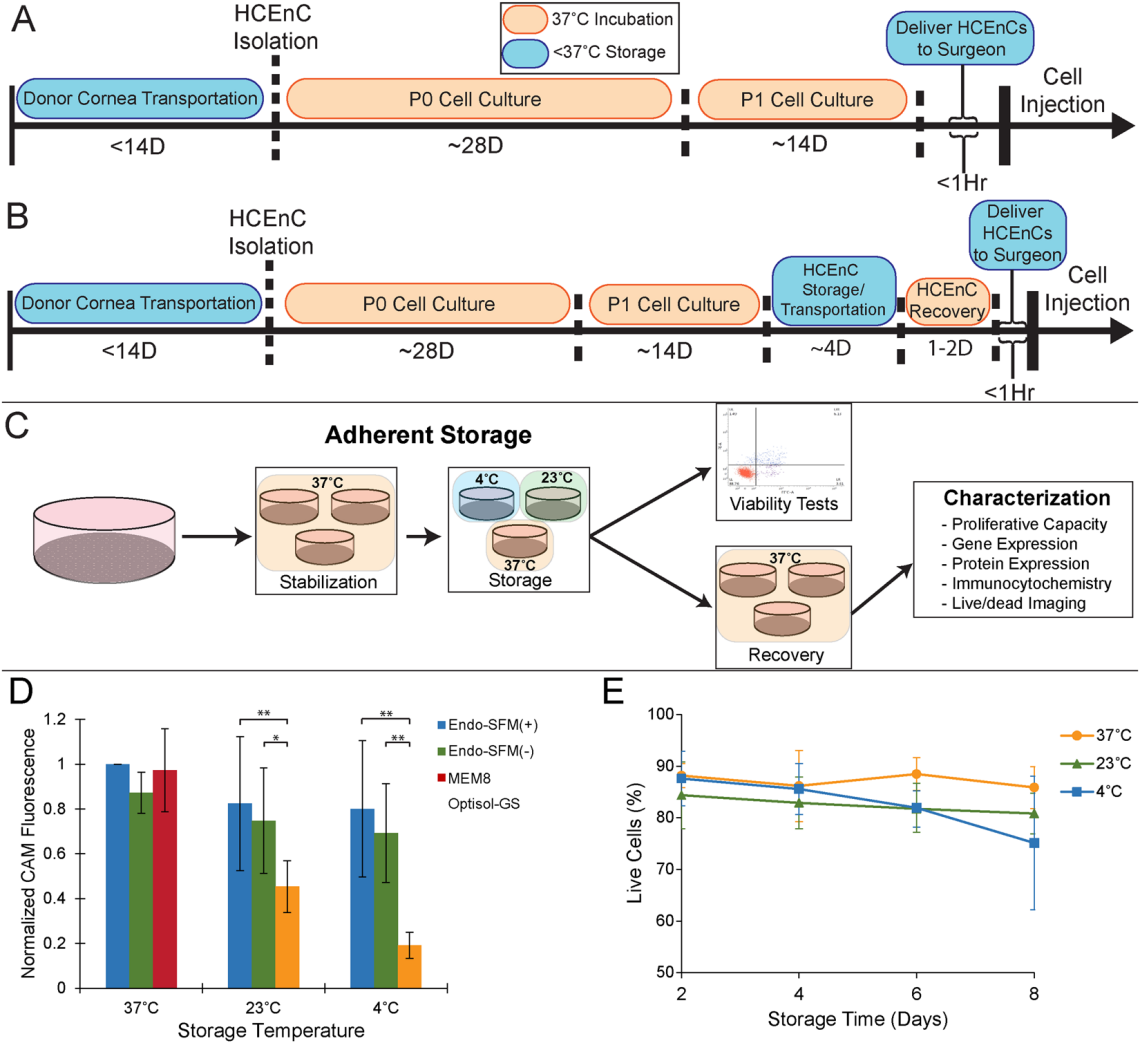
Optimization of hypothermic storage protocol for corneal endothelial cells. (**A**) Current HCEnC culture protocols necessitate delivery of cells from the laboratory directly to the surgeon in a short time frame, while hypothermic storage (**B**) would create a window for storage/transport of cells. (**C**) To mimic an HCEnC-seeded scaffold, cells were initially stored as adherent monolayers in tissue culture dishes. Following storage, cells were processed directly to assess viability or returned to the incubator for 2 days of recovery before further analysis. (**D**) To determine the optimum storage medium, HCEnC viability was assessed with calcein AM (CAM) fluorescence after 2 days in storage, without any recovery at 37 °C. Multiple culture media were tested, including Endo-SFM with serum (Endo-SFM(+)) and without serum (Endo-SFM(-)) as well as Optisol-GS and an MEM-based organ culture medium with 8% serum. All fluorescence values were normalized to Endo-SFM(+) at 37 °C. Statistical significance was detected between storage in Optisol and Endo-SFM with and without serum at both 4 °C and 23 °C (*p < 0.05, **p < 0.01; n = 4). (**E**) Viability in Endo-SFM(+) over an extended hypothermic storage time was assessed using an Annexin V/propidium iodide flow cytometry assay. (n = 3).

We sought to define appropriate conditions for hypothermic preservation of HCEnC that could be integrated into our existing protocol for cell injection (Fig. 1A). Optimization experiments were carried out using adherent HCEnCs (Fig. 1B) to allow for monitoring of cell morphology during storage. A comparison of post-storage viability demonstrated that Human Endothelial-SFM was superior to Optisol-GS at both 23 °C and 4 °C (Fig. 1C; n = 4). The presence of 5% serum did not benefit cellular viability in this model. However, due to its known importance for HCEnC functionality in culture, which could have an impact on *in vivo* functionality beyond simple cell survival^5,17^, we elected to use Endo-SFM(+) as our storage media in subsequent experiments. Assessment of storage duration on HCEnC viability demonstrated no significant effect of preservation temperature at each time point (Figs. 1D,E; n = 3).

### Adherent storage morphology

**Figure 2:**
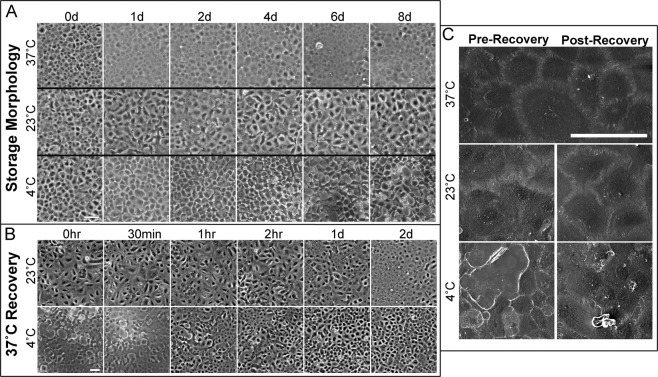
Corneal endothelial cell morphology during hypothermic storage in Endo-SFM(+) and subsequent recovery. (**A**) Representative phase-contrast micrographs demonstrate morphological changes during storage and (**B**) during recovery at 37 °C. (Scale bar: 100 μm) (**C**) These morphological changes are also shown in scanning electron microscopy micrographs. (Scale bar: 100 μm).

Although temperature did not have a significant effect on the viability of HCEnCs in Endo-SFM, marked morphological changes were apparent (Fig. 2A). HCEnCs maintained at 37 °C retained a regular, hexagonal monolayer. However, HCEnCs at 23 °C demonstrated blurred intercellular boundaries, whilst HCEnCs at 4 °C adopted a retracted appearance with narrow projections. Interestingly, these morphological changes rapidly reverted when cells were returned to 37 °C (Fig. 2B,C). Therefore, we employed a 48-hour recovery period at 37 °C in our adherent storage model for characterization experiments. In addition, a 4-day storage duration was used for further experiments, as this time frame is believed to be sufficient for transportation around the globe.

### Adherent storage viability and proliferative capacity

**Figure 3:**
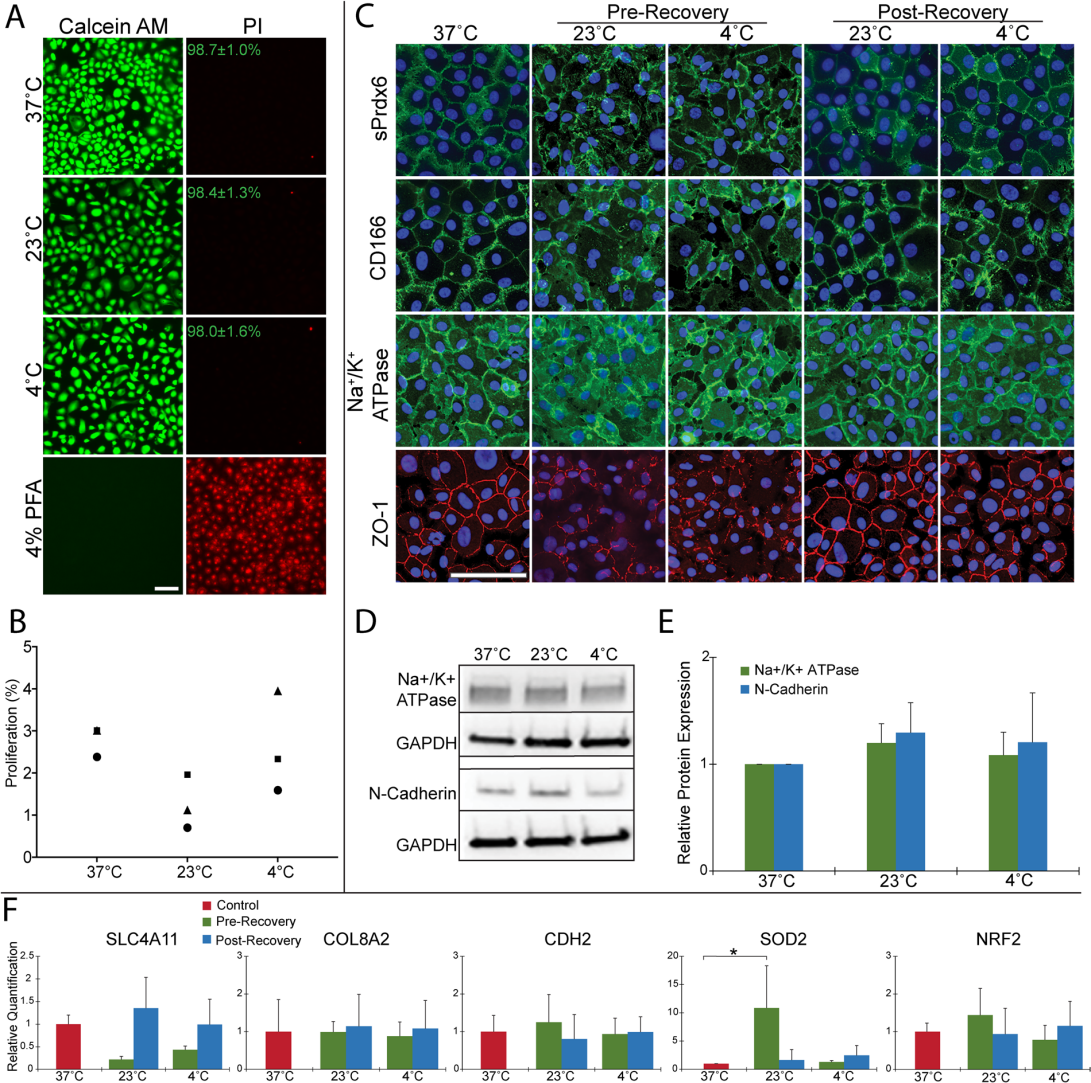
Characterization of post-adherent storage endothelial cells using a 4-day storage period in Endo-SFM(+) with 48-hour recovery at 37 °C. (**A**) Live/dead staining was performed on HCEnCs following storage and recovery. Mean ± SD percentage of live cells is noted for each temperature. (n = 3; Scale bar: 100 μm) (**B**) HCEnCs were re-seeded after recovery and assessed using a Click-iT EdU kit to determine proliferation rate. Each marker represents cells from a single donor. (n = 3) (**C**) Expression of surface markers Na^+^/K^+^ ATPase, ZO-1, cell surface peroxiredoxin 6 (sPrdx6), and CD166 was assessed with immunocytochemistry of post-storage HCEnCs, performed both before and after recovery at 37 °C. (Scale bar: 100 μm) (**D,E**) Western blot analysis and quantification of HCEnC protein markers Na^+^/K^+^ ATPase and N-cadherin relative to GAPDH and normalized to 37 °C expression levels. (n = 3). The panels show cropped images obtained from a single western blot membrane. A full-length blot is presented in supplementary Fig. 1. (**F**) Quantitative PCR analysis of mRNA extracted from HCEnCs before and after recovery was performed. Relative quantification values were determined using a GAPDH internal control, and experimental gene expression values of SLC4A11, COL8A2, CDH2, SOD2, and NRF2 were normalized to the 37 °C control. (n = 3; *p < 0.05).

Since delayed cell death following rewarming has been previously documented^40,48^, we sought to confirm that post-storage HCEnCs maintain viability after recovery at 37 °C. Live/dead imaging demonstrated preserved viability of HCEnCs from each storage temperature, with average viability > 98% across conditions (Fig. 3A; n = 3). Although proliferation rate was lower among HCEnCs from 23 °C at 1.3 ± 0.6% compared to 2.8 ± 0.4% at 37 °C or 2.6 ± 1.2% at 4 °C, it was not statistically significant (Fig. 3B; n = 3).

### Adherent storage endothelial marker expression

In addition to the morphological differences described above, storage at 4 °C and 23 °C caused irregular expression and staining patterns of endothelial surface markers Na^+^/K^+^ ATPase, ZO-1, cell-surface peroxiredoxin 6 (PRDX-6), and CD166 (Fig. 3C). However, marker expression returned to normal after the 37 °C recovery (Fig. 3C). Western blot analysis confirmed that post-storage cells express Na^+^/K^+^ -ATPase. In addition, expression of the HCEnC marker N-cadherin was not significantly affected post-storage (Fig. 3D,E; n = 3). Expression of corneal endothelial-associated genes COL8A2^49^ and CDH2 (N-cadherin)^50^ were unchanged throughout storage and recovery. Although SLC4A11^49^ demonstrated a non-significant decrease during storage at both 4 °C (0.43 ± 0.08-fold) and 23 °C (0.22 ± 0.07-fold) this was rescued following recovery (0.99 ± 0.56-fold 4 °C, 1.35 ± 0.69-fold 23 °C) (Fig. 3F; n = 3). Expression of oxidative stress response gene NRF2 was also unchanged. However, expression of superoxide dismutase 2 (SOD2) levels were elevated in the pre-storage 23 °C group (10.9 ± 7.46-fold) (Fig. 3F).

### Suspension storage protocol optimization

**Figure 4:**
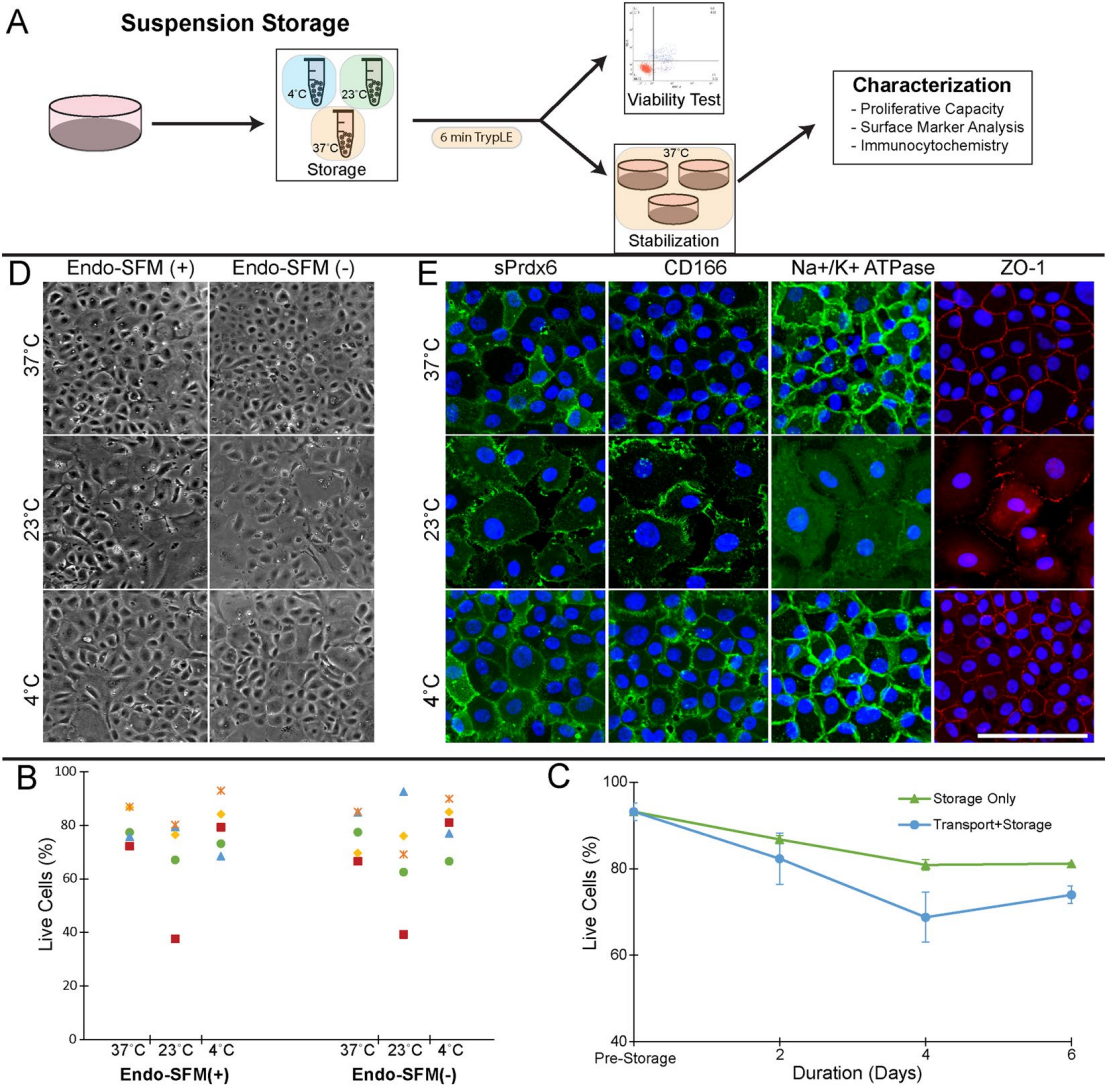
Optimization of hypothermic suspension storage for cultured endothelial cells. (**A**) To facilitate easier transportation and handling prior to cell injection therapy, HCEnCs were suspended in storage media within sealed Eppendorf tubes and stored at various temperatures. Following brief treatment with TE to dissociate cell clumps, cells were either analyzed immediately to assess viability, or seeded onto a tissue culture dish for further characterization. (**B,C**) Phase-contrast micrographs and immunocytochemistry images of HCEnCs stored for 2 days and subsequently re-seeded and maintained at 37 °C. All immunocytochemistry images were acquired from HCEnCs stored in Endo-SFM with 5% serum (Endo-SFM(+)). (Scale bar: 100 μm) (**D**) To assess the effect of suspension storage media and temperature on viability, cells were suspended in Endo-SFM(+) and Endo-SFM without serum (Endo-SFM(-)) at 4 °C, 23 °C, and 37 °C. After 2 days in storage, viability was assessed with an Annexin V/propidium iodide flow cytometry assay. (n = 5) (**E**) To assess the effect of storage duration, cells were stored in Endo-SFM(+) at 4 °C and viability was assessed with Annexin V/propidium iodide at several time points (pre-storage, 2 days, 4 days, and 6 days). HCEnCs were either placed directly into storage, or subject to simulated transportation on a shaker plate for the first 2 days. Statistical significance was determined via Dunnett’s post-hoc test with pre-storage viability as the control. (*p < 0.05, **p < 0.01; n = 3).

Since transportation of adherent cells is logistically challenging, we tested a suspension storage protocol. Single cell suspensions of HCEnCs in Endo-SFM storage media were place in sealed Eppendorf tube and maintained at either, 4 °C, 23 °C, or 37 °C (Fig. 4A). Post-storage HCEnCs were either processed immediately to assess viability or, re-seeded onto tissue culture dishes for further characterization (Fig. 4A). Morphological analysis of post-storage HCEnCs demonstrated hexagonal morphology after preservation in 37 °C and 4 °C, while cells kept at 23 °C appeared more irregular (Fig. 4B). Immunohistochemistry demonstrated abnormal staining and morphology of HCEnCs stored at 23 °C compared to 37 °C and 4 °C (Fig. 4C). Viability analysis showed no significant differences based on storage temperature or inclusion of serum in the storage media (Fig. 4D; n = 5). Because of the abnormal morphology and marker expression associated with 23 °C storage and the logistical difficulties of 37 °C storage, we selected 4 °C as the ideal suspension storage temperature. Endo-SFM(++) was used as the suspension storage media for subsequent experiments, both to maintain consistency with our adherent storage model and to mimic the supportive environment of our *in vitro* culture system^17,20,51^.

We evaluated the effect of storage duration and transport conditions on HCEnC viability. Storage-only tubes were placed directly into a 4 °C container, while the transport+storage tubes were subject to simulated transportation on a 60 revolutions per minute shaker plate at 4 °C for the first 2 days of the experiment. Although there was no significant difference between the pre-storage viability (89.9 ± 5.9%) and either experimental condition at 2 days, a significant decrease was present after 4 days (63.7 ± 9.7%) and 6 days (74.6 ± 1.0%) in the transport+storage condition. (Fig. 4E; n = 3). We therefore used a 2-day storage duration for subsequent experiments. Of note, simulated transportation was not used for these later experiments, as we wished to first characterize post-storage HCEnCs in optimal conditions before testing the effects of additional stressors like transport.

### Suspension storage characterization

**Figure 5:**
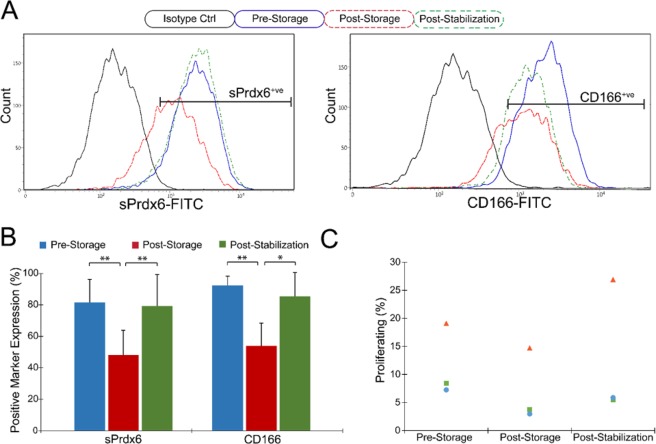
Characterization of HCEnCs stored in 4 °C Endo-SFM(+) suspension. (**A,B**) HCEnCs were collected and analyzed with flow cytometry for expression of cell surface Prdx6 (sPrdx6) and CD166 before storage, immediately after storage, and after 2 days stabilization at 37 °C. Staining with an IgG mouse isotype antibody served as a negative control. Positive expression gates were set to include < 2% of isotype controls. (*p < 0.05, **p < 0.01; n = 4) (**C**) HCEnCs were re-seeded pre-storage, post-storage, and post-stabilization and processed with a Click-iT EdU kit to assess proliferative capacity (n = 3).

In order to assess HCEnC quality after suspension storage, we analyzed cells immediately before and after storage, and following re-seeding for a 48 hour stabilization period at 37 °C. Expression of endothelial surface markers PRDX-6 and CD166 was significantly diminished in the post-storage group (48.2 ± 15.7% PRDX-6^+ve^; 53.9 ± 14.5% CD166^+ve^) compared to the pre-storage group (81.5 ± 14.6%; 92.4 ± 5.8%), but recovered following post-stabilization (79.2 ± 20.2%; 85.5 ± 15.1%) (Fig. 5A,B; n = 4). A similar effect was demonstrated with regard to proliferation rate, which demonstrated a non-significant decrease from pre-storage (11.6 ± 6.5%) to post-storage (7.1 ± 6.6%) but recovered following stabilization (12.7 ± 12.3%) (Fig. 5C; n = 3).

### Functionality of HCEnCs post hypothermic storage

**Figure 6:**
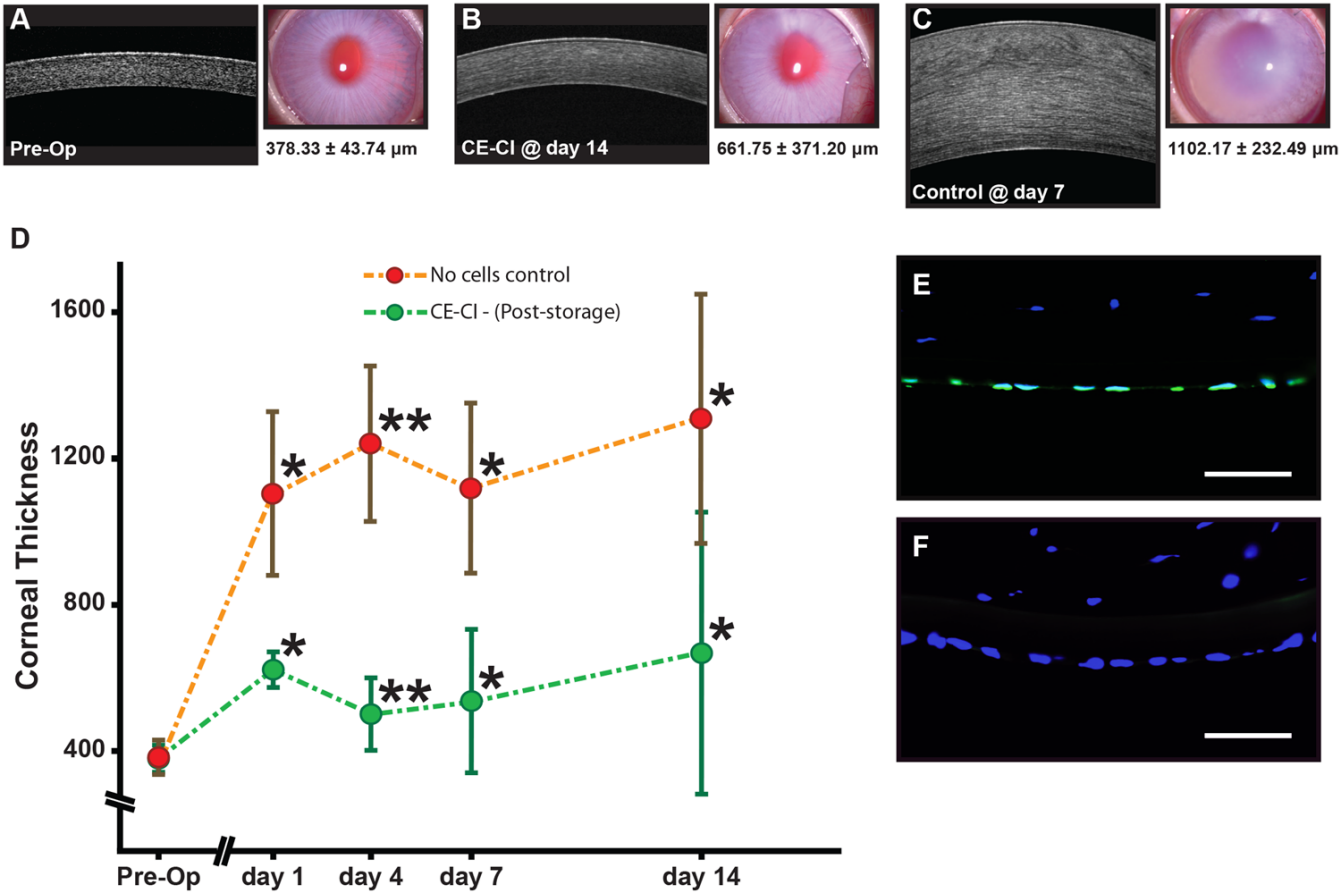
Functional assessment of HCEnCs post storage in a rabbit model of bullous keratopathy. Representative *in vivo* confocal and corresponding corneal images of (**A**) pre-operated rabbit, (**B**) rabbit receiving CE-CI at day 14, and (**C**) control rabbit with no cells injected at day 7. (**D**) Graph summarizing the corneal thickness of rabbits that received CE-CI of HCEnCs post-storage (n = 4) and controls (n = 4) over 14 days (*p < 0.05, **p < 0.01). Staining of a human-specific nuclei antibody on (**E**) a section of an excised cornea of rabbit receiving CE-CI of HCEnCs post-storage, and (**F**) the rabbit cornea as a negative control. Scale bar: 50 μm.

Using a CE-CI rabbit model of bullous keratopathy as previously described^52^, we assessed the functional capacity of HCEnCs, following 48 hours storage in hypothermic suspension. Corneas of rabbits receiving CE-CI from post-stored HCEnCs were significantly thinner throughout the 14 days follow-up period. However, the cornea of rabbits in the control group were thicker (Fig. 6A–D), as supported by representative *in vivo* confocal images and their corresponding slit-lamp corneal images of pre-operative cornea, rabbit receiving CE-CI at day 14, and control rabbit without any cells injected at day 7 (Fig. 6A–D; *P < 0.05 **P < 0.005). No significant IOP variation or elevation was observed in any of the post-operative eyes (results not shown). The mean corneal thickness of rabbits that underwent CE-CI elevated to 618.75 μm ± 49.88 μm on day 1, which was in stark contrast to the corneal thickness of control rabbits 1093.42 μm ± 225.62 μm (Fig. 6; Table 1; *P < 0.05). The mean corneal thickness of rabbits receiving CE-CI were significantly lower (Table 1), and were observed to be 500.67 μm ± 104.47 μm at Day 4**; 540.92 μm ± 202.10 μm at Day 7*; and 661.75 μm ± 371.20 μm at Day 14*, compared to average corneal thickness of control rabbits 1230.42 μm ± 212.23 μm at Day 4; 1102.17 μm ± 232.49 μm at Day 7; and 1289.42 μm ± 337.17 μm at Day 14 (Fig. 6D; Table 1; *P < 0.05 **P < 0.005). Finally, we assessed the specificity of the human-specific nuclei antibody of the excised rabbit cornea that received CE-CI and showed that it only labeled the nuclei of corneal endothelial layer indicating that it the functionality observed was the result of the injected HCEnCs (Fig. 6E). Neither the rabbit stromal keratocytes (Fig. 6E), nor the rabbits’ endogenous corneal endothelium was not labelled by the human-specific nuclei antibody (Fig. 6F).

**Table 1:** Averaged corneal thickness of rabbits receiving CE-CI of HCEnCs following post hypothermic storage and control rabbits *P < 0.05; **P < 0.005.

Time	Average Corneal Thickness (μm ± SD)	
	CE-CI (n = 4)	Control (n = 4)
Pre-Operative	378.33 ± 43.74	371.67 ± 16.68
Day 1	618.75 ± 49.88^*^	1093.42 ± 225.62^*^
Day 4	500.67 ± 104.47^**^	1230.42 ± 212.23^**^
Day 7	540.92 ± 202.10^*^	1102.17 ± 232.49^*^
Day 14	661.75 ± 371.20^*^	1289.42 ± 337.17^*^

## Discussion

The cornea is by far the most transplanted organ in the world, with over 184,000 transplant operations in 2012 compared to approximately 127,000 transplantations of all other solid organs combined in 2015^10,53^. Despite this considerable surgical volume, there remains a growing unmet need for donor corneas^10^. Endothelial dysfunction is one of the primary indications for corneal transplantation, responsible for more than 39% of transplants performed globally^10^. While allogeneic transplantation is the current standard treatment, endothelial cellular therapy is an emerging alternative option. The first-in-man clinical trial of cell injection therapy by Kinoshita *et al*. reported considerable improvements in corneal edema and visual acuity that persisted over a 2-year follow-up. This demonstrated that corneal endothelial cell therapy is a viable approach in humans^26^. However, that study and prior reports on HCEnC therapy used cells cultured in GMP laboratories which are limited to a small number of large academic medical centers^17,26^. Wider implementation of endothelial cell therapy will therefore require methods of transporting HCEnCs from the laboratory to clinical environments. Since preservation in a 37 °C, 5% CO_2_ incubator is incompatible with shipping logistics, interest has emerged in hypothermic HCEnC storage.

Currently in the US, donor corneas are shipped in Optisol-GS at 2–8 °C^35,36^. This creates a 7 day window for transplantation, with a very minor reduction in post-transplant HCEnC density and graft survival among corneas in the 8–14 day range^35,36^. However, earlier studies have shown that Optisol poorly maintains *in vitro* HCEnC viability and morphology in hypothermic conditions^37,40^, which may be attributable to significant changes in gene expression that occurs with HCEnC culture^39^. Our results confirm these observations, as Optisol-GS performed significantly poorer than Endo-SFM at both 23 °C and 4 °C. While Endo-SFM has largely failed to supplant MEM-based media for organ culture of donor corneas^54–56^, our study shows that it deserves consideration as a hypothermic storage medium.

Although media supplementation with serum was critical for *in vitro* cultivation of HCEnCs^5,17,20^, we hypothesized that it may not be as important during hypothermic storage when cell metabolism is reduced. This was confirmed for both adherent and suspension HCEnC storage, with no observed difference in viability between cells stored with and without 5% serum. Because of the possible presence of serum affecting HCEnC functionality without disturbing viability, we elected to keep 5% serum in the storage medium for subsequent experiments. However, removal of serum would also eliminate a potential source of microbial contamination^57^, and thus could be considered, particularly if future studies confirm that serum is non-essential for short-term hypothermic HCEnC storage.

We investigated both adherent and suspension storage models in this study, each of which was appropriate for different situations. A major advantage of the adherent model is that cellular morphology can be observed in real-time. Morphological changes in HCEnCs can often occur in conjunction with loss of function, as seen with the development of large vacuoles in senescent cells or the conversion to an elongated, fibroblastic morphology during endothelial-mesenchymal transition (EMT)^5^. Furthermore, HCEnCs are normally attached to the DM *in vivo*, and an adherent storage system best replicates this native environment. However, although acceptable for laboratory investigation, maintaining HCEnCs in a sealed tissue culture dish is likely to be impractical for transportation. Excessive movement of the shipping container could lead to media spillage and subsequent cell death. While this model may be compatible with an HCEnC-seeded scaffold, which could be attached to a polyester membrane carrier and immersed in storage medium as seen with limbal epithelial cell grafts^58^, it appears inappropriate for cell injection therapy. For this reason, we employed a suspension model in which dissociated HCEnCs were stored in a sealed Eppendorf tube. These cells could potentially be re-suspended in fresh media by the receiving surgeon and injected directly from the shipping tube. However, our results demonstrated improved endothelial surface marker expression after a 48-hour adherent stabilization period in 37 °C compared to cells taken directly from 4 °C storage. This improvement could be due to several factors, including the elimination of non-functional cells that failed to adhere to the tissue culture surface or cellular recovery when removed from the stresses of hypothermic storage.

One of the most notable changes we observed with hypothermic storage is alteration of endothelial morphology. HCEnCs at 23 °C demonstrated a blurring of intercellular boundaries, while cells at 4 °C appeared retracted, consistent with prior reports in porcine CEnCs^40^. Interestingly, these changes appear to be reversible when HCEnCs are returned to 37 °C. The increase in SOD2 expression in pre-recovery HCEnCs at 23 °C supports prior reports that these temperature-mediated changes are due to oxidative stress^40,59^. SOD2, also known as manganese-dependent superoxide dismutase, catalyzes the conversion of highly active superoxide molecules into oxygen and hydrogen peroxide in order to regulate reactive oxygen species (ROS) levels^60^. Studies in rabbit corneal endothelium have demonstrated that SOD2 expression increases in a stress response to phacoemulsification^61^, while SOD2 knockout increases both ROS levels and the rate of apoptosis^62^. Further investigations are necessary to determine the specific role that increased SOD2 antioxidant capacity serves in hypothermic HCEnCs.

As mentioned above, preservation of donor corneas in Optisol at 2–8 °C maintained endothelial function for 7–14 days. While storage of cultured HCEnCs for a similar time frame would be ideal, one major benefit of cultured cells is that they can be stored in 37 °C incubator for an extended length of time, with excellent maintenance of cell quality. As such, HCEnCs could be kept in the laboratory incubator until requested by a surgeon, at which point they would be transported in hypothermic conditions to the host institute for cellular preparation prior before cellular therapy. The cells would therefore only need to survive for the length of transportation, in addition to a nominal storage time, and preparation at the recipient site. The 4-day and 2-day storage durations used for our adherent and suspension models respectively should be sufficient to reach most destinations across the globe.

A recent report by Bartakova *et al*. described a storage system in which HCEnCs were suspended in saline and transported at 4 °C followed by storage at 23 °C^45^. Their data demonstrated that post-storage cells retained viability and express the function-associated marker, CD56. We sought to characterize our post-storage cells in greater detail to justify their use in cell therapy. The *in vitro* experiments we performed demonstrated that HCEnCs cells retain a proliferative capacity and continue to express endothelial markers Na^+^/K^+^ ATPase, ZO-1, sPrdx6, CD166, and N-cadherin after storage. While these are encouraging results, ultimately the most important criteria of HCEnCs in cellular therapy is their ability to maintain corneal deturgescence *in vivo*. To this end we demonstrated for HCEnCs stored in a hypothermic suspension for 2 days are functionally in a rabbit model of bullous keratopathy. Future work will include the assessment of post-storage HCEnC function for adherent preparation of tissue engineered graft material^17^.

In conclusion, we have provided data to support our hypothesis that functional HCEnCs can be preserved in hypothermic conditions, specifically with the use of Endo-SFM as a storage medium. Both adherent cells and those in suspension can be preserved at 4 °C, while only adherent HCEnCs should be stored at 23 °C. Post-stored cells demonstrate similar characteristics, including, proliferative capacity, gene expression, and surface marker expression to those cells maintained at 37 °C. As endothelial cell therapy approaches clinical reality, this method of hypothermic storage may serve an essential role in assimilating such new technology into existing infrastructure.

## Methods

### Materials

Ham’s F12, Medium 199, Human Endothelial-SFM, Minimum Essential Medium (MEM), Dulbecco’s Phosphate-Buffered Saline (PBS), Insulin/Transferrin/Selenium (ITS), TrypLE Express (TE), 1 M HEPES buffer, and antibiotics, were all purchased from Life Technologies (Carlsbad, CA, USA). Trypan blue (0.4%), alizarin red, paraformaldehyde (PFA), Triton X-100, Collagen IV from human placenta, and ascorbic acid were purchased from Sigma (St. Louis, MO, USA). Calcein AM, propidium iodide (PI), Hoechst 33342, and PureLink RNA Micro kit were obtained from Invitrogen (Carlsbad, CA, USA). Recombinant basic fibroblast growth factor (bFGF, R&D, Minneapolis, MN, USA). Rho-associated, coiled-coil protein kinase (ROCK) inhibitor, Y-27632 (Miltenyi Biotec, Bergisch Gladbach, Germany). FNC coating mixture (United States Biologicals, Swampscott, MA, USA). Liberase TH (Roche Mannhein, Germany). EquaFetal serum was obtained from Atlas Biologicals (Fort Collins, CO, USA). Optisol-GS was purchased from Basuch & Lomb (Rochester, NY, USA).

### Research-grade human corneoscleral tissues

This study was approved by Singhealth centralized institutional review board (Ref: 2015/2320 and 2017/2412). All research-grade, human cadaver corneal tissues were procured through Lions Eye Institute for Transplant and Research (Florida, USA) and Miracles in Sight (North Carolina, USA). Informed consent was obtained from the next of kin for all deceased donors and adhered to the principles outlined in the Declaration of Helsinki. A total of 15 pairs of donor corneal tissues ranged from 3 to 36 years old with endothelial cell count of at least 2000 cells per mm^2^ were procured for the culture of human HCEnCs (Table 2). Corneoscleral tissues were preserved in Optisol-GS at 4 °C and processed within 14 days of preservation.

**Table 2:** Primary HCEnC donor information.

Serial Number	Age	Sex	Days to Culture	Cell Count (OS/OD)	Cause of Death	Figure					
						1	2	3	4	5	6
01	3	M	9	3817/3774	Blunt Force Trauma	●	●				
02	9	M	9	3236/-	Blunt Force Trauma	●	●	●			
03	36	M	6	3289/2915	MVA	●	●	●			
04	36	M	9	2933/-	GSW-Head	●		●		●	
05	35	M	18	2646/2681	Suicide	●	●	●			
06	20	M	12	2915/2882	MVA		●		●		
07	27	M	12	3049/2817	Overdose		●	●			
08	33	M	9	3333/3175	Melanoma			●	●		
09	34	M	11	3279/3247	Aspiration			●	●		
10	22	F	8	2762/2688	Sub Arachnoid Hemorrhage				●		
11	29	F	11	2392/2591	MVA				●		
12	32	M	12	2967/2933	MVA			●	●		
13	17	M	9	3040/3300	Acute Cardiac Crisis					●	
14	22	M	11	3506/3448	Coronary Artery Disease					●	
15	11	M	8	3135/3344	Respiratory Failure				●	●	
16	18	M	16	3636/3484	GSW-Head						●
17	8	M	7	3236/3236	MVA						●
18	18	F	7	2865/2933	Sub Arachnoid Hemorrhage						●
19	24	F	10	3115/2899	Overdose						●

### Cell isolation and culture

Primary HCEnCs isolated from pairs of donor corneas were propagated using a dual media system as previously described^17,20^. Briefly, HCEnCs were isolated from peeled Descemet’s membrane (DM) by Liberase digestion (up to 4 hours) followed by dissociation with TE. Isolated cells were washed twice before being seeded onto Collagen IV-coated culture vessels in maintenance medium (M5-Endo; Human Endothelial-SFM (Endo-SFM) supplemented with 5% EquaFetal serum) overnight. HCEnCs were subsequently cultured in proliferative medium (M4-F99; Ham’s F12/M199, 5% serum, 20 μg/ml ascorbic acid, 1x ITS, and 10 ng/ml bFGF). Unless otherwise stated, media was supplemented with the ROCK inhibitor Y-27632 (10 μm). When HCEnCs reached 80% to 90% confluency, media was changed to maintenance media (M5-Endo) for a further two days. HCEnCs were sub-cultured via single-cell dissociation using TE and plating at a density of approximately 1 × 10^4^ cells per cm^2^ on pre-coated plates. All cultures were incubated in a humidified atmosphere at 37 °C and 5% CO_2_. HCEnCs at the second and third passage were used for experiments.

### Hypothermic storage

Storage media for adherent HCEnCs consisted of M5-Endo supplemented with 12.5 mM HEPES buffer, with 5% serum (Endo-SFM(+)) or without serum (Endo-SFM(-)). Herein, M5-Endo is used to refer to our *in vitro* maintenance medium without HEPES, while Endo-SFM (+/-) is used to refer to our storage medium with HEPES. Parafilm sealed dishes were stored at either 37 °C, 23 °C, or 4 °C. For post-storage recovery, HCEnCs were returned to a 37 °C incubator for 48 hours. A Nikon TS1000 phase contrast microscope with a Nikon DS-Fi1 digital camera (Tokyo, Japan) was used to capture cellular morphology.

For hypothermic suspension storage, HCEnCs suspended in storage media were aliquoted into 1.5 mL Eppendorf tubes and sealed with Parafilm. Following storage for 48 hours at either 37 °C, 23 °C, or 4 °C, HCEnCs were treated with TE for 6 minutes at 37 °C to dissociate cell clusters. Cells were either seeded in tissue culture plates in M5-Endo or processed immediately. ROCK inhibitor was excluded from the storage medium, as we did not want to promote cell attachment in this model. To simulate transportation, tubes were placed on a shaker rack set to 60 revolutions per minute at 4 °C.

### Assessment of HCEnC viability

HCEnCs were seeded and maintained in M5-Endo for 24 hours. Medium was replaced with either: Endo-SFM(+), Endo-SFM(-), Optisol-GS, and a MEM-based culture medium (MEM, 12.5 mM HEPES, 250 ng/mL amphotericin B, 100 Units/mL penicillin/streptomycin, and 8% serum) and incubated for 2 days. HCEnCs were incubated with 2 μM calcein AM at 37 °C for 30 minutes. Fluorescence was measured with a microplate fluorometer (Tecan Infinite M200 Pro, Tecan Systems, CA, USA) with an excitation/emission filter of 485 nm/535 nm. Background fluorescence, measured in wells containing calcein AM without cells, was subtracted from all values.

A Fluorescein isothiocyanate (FITC) Annexin V apoptosis detection kit with propidium iodide (BioLegend, San Diego, CA, USA) was also used to assess HCEnC viability in both adherent and suspension storage. Briefly, washed and dissociated HCEnCs were re-suspended and incubated in solution containing Annexin V-FITC and PI following the manufacturer’s instructions. Cells were analyzed via flow cytometry using a FACS Verse flow cytometer (Becton Dickinson, East Rutherford, NJ, USA) as previously described^17,19^.

To confirm cell viability after hypothermic storage and recovery at 37 °C, live/dead imaging was performed with calcein AM and propidium iodide (PI). Post-recovery HCEnCs were washed with PBS and incubated in 2 μM calcein AM and 4 μM PI for 30 minutes at 37 °C. Negative control consisted of HCEnCs fixed with 4% PFA for 10 minutes. Fluorescent staining was visualized with a Nikon C2+fluorescent microscope (Tokyo, Japan).

### Scanning electron microscopy

HCEnCs were seeded on FNC-coated glass coverslips and maintained in M5-Endo for at least 48 hours. Following a four-day storage, coverslips were removed and fixed with neutrally buffered 2% glutaraldehyde (Electron Microscopy Sciences, Hatfield, PA, USA) at 4 °C for 4 hours. Alternatively, samples were placed at 37 °C for 48 hours recovery, and subsequently fixed. Samples were processed as previously described^17^. Briefly, samples were post-fixed in 1% osmium tetroxide at room temperature for 1 hour. The samples were then dehydrated, critical point dried (BALTEC, Balzer, Liechtenstein) and mounted onto a metal stub using carbon adhesive tabs. Samples were sputter-coated for 160 seconds with a 22 nm layer of gold-phalladium alloy (BALTEC) and examined under a scanning electron microscope (Quanta 650FEG; FEI, Hillsboro, OR, USA).

### Immunocytochemistry

For adherent storage, HCEnCs were seeded at a density of 1000 cells/mm^2^ on FNC-coated glass coverslips and maintained in M5-Endo for at least 48 hours, followed by 4 days in storage and 48 hours recovery at 37 °C. Cells were fixed in either 4% paraformaldehyde or, 100% ice-cold ethanol for 5 minutes at 4 °C (Table 3). Following blocking in 10% normal goat serum samples were incubated with primary antibodies at room temperature for 1 hour (Table 3). Following washing in PBS, samples were labeled with the appropriate secondary antibodies: AlexaFluor 488 conjugated goat anti-mouse IgG or AlexaFluor 546 conjugated goat anti-rabbit IgG (Life Technologies, Thermo Fisher Scientific) at 2.5 μg/ml for 1 hour at room temperature in the dark. Samples were washed and mounted in Vectashield containing DAPI (Vector Laboratories, Burlingame, CA, USA), and visualized using a Zeiss Axioplan 2 fluorescence microscope (Carl Zeiss, Oberkochen, Germany).

**Table 3:** List of primary antibodies in this study. IF, immunofluorescence; WB, Western blot; FACS, fluorescence activated cell sorting.

Antibody (Clone)	Company (Catalog #)	IF Fixative	Conc (IF)	Conc (WB)	Conc (FACS)
Na^+^/K^+^ ATPase (0.T.1)	Santa Cruz (sc-71638)	100% Ethanol, 5 min, 4 °C	5 μg/mL	200 ng/mL	N/A
ZO-1 (Polyclonal)	Invitrogen (40–2200)	100% Ethanol, 5 min, 4 °C	5 μg/mL	N/A	N/A
CD-166 (TAG-1A3)	In-house^63^	4% PFA 10 min, room temp	Supernatant	N/A	Supernatant
sPrdx6 (TAG-2A12)	In-house^63^	4% PFA 10 min, room temp	Supernatant	N/A	Supernatant
N-cadherin (8C11)	BD Pharmingen (561553)	N/A	N/A	500 ng/mL	N/A
GAPDH (FF26A/F9)	Biolegend (649202)	N/A	N/A	500 ng/mL	N/A
Mouse IgG Isotype (MG1–45)	Biolegend (401402)	N/A	N/A	N/A	1.2 μg/mL
Human Nuclei (Clone 235–1)	Merck Millipore	4% PFA 10 min, room temp	1:100	N/A	N/A

For immunocytochemistry following suspension storage, post-storage cells were seeded onto glass coverslips, maintained in M5-Endo for at least 48 hours, and then processed for staining as described above.

### Gene expression analysis

Total RNA was extracted from post-storage HCEnCs using the PureLink RNA Micro kit with RNase-free DNase treatment (Qiagen, Hilden, Germany). RNA was reverse transcribed using the High-Capacity cDNA Reverse Transcription kit (Thermo Fisher Scientific, MA, USA). Quantitative PCR was carried out on a Lightcycler 480 system (Roche, Basel, Switzerland), using TaqMan gene expression assays (Table 4) and TaqMan fast master mix (Thermo Fisher). Gene expression levels were normalized with endogenous levels of *GAPDH* (glucose 6-phosphate dehydrogenase) and relative fold changes were analyzed using the ΔΔCt method.

**Table 4:** List of probes used for quantitative PCR in this study.

Gene Name	TaqMan Probe
Glyceraldehyde 3-phosphate dehydrogenase (GAPDH)	Hs02758991_g1
Solute carrier family 4 member 11 (SLC4A11)	Hs00984689_g1
Collagen type VIII alpha 2 chain (COL8A2)	Hs00697025_m1
N-cadherin (CDH2)	Hs00983056_m1
Superoxide dismutase 2 (SOD2)	Hs00167309_m1
Nuclear factor related factor 2 (NRF2/NFE2L2)	Hs00975961_g1

### Western blot

Following storage, HCEnCs were lysed in Laemmli SDS sample buffer. Lysates were separated using Mini-PROTEAN gel systems (BioRad), transferred to a polyvinylidene difluoride (PVDF) membrane and probed with antibodies as specified in Table 3. Quantification was performed using ImageLab software (BioRad).

### Cell proliferation assay

Proliferation rates of post-storage HCEnCs was assessed using the EdU incorporation Click-iT assay (Life Technologies), according to the manufacturer’s instructions and as previously described^19^. For adherent HCEnCs, after storage they were maintained at 37 °C for 48 hours to allow recovery. Following which, they were passaged and seeded onto FNC-coated glass slides at a density of 5 × 10^3^ cells/cm^2^ in M4-F99 proliferative medium for 24 hours. For suspension storage, donor matched HCEnCs were similarly seeded onto glass slides at three time points. 1) prior to storage, 2) immediately following storage, and 3) after 48 hours post-storage stabilization at 37 °C. Cells were further incubated in M4-F99 containing 10 μm EdU for an additional 24 hours. Samples were fixed in 4% PFA for 15 minutes, permeabilized with 0.1% Triton X-100 in PBS for 20 minutes and incubated in the Click-iT reaction cocktail for 30 minutes. Samples were examined with a Zeiss Axioplan 2 fluorescence microscope (Carl Zeiss, Germany). At least 300 nuclei were examined for each experimental set.

### Flow cytometry

HCEnCs were dissociated and resuspended in 2% BSA in PBS. Cells were incubated with primary antibody or isotype control (Table 3) for 25 minutes, washed with 2% BSA, and subsequently incubated with AlexaFluor 488 goat anti-mouse antibody (1.5 μg/mL, Life Technology) for 25 minutes. All incubations were performed on ice. Analysis was then carried out with a FACS Verse flow cytometer (Becton Dickinson, East Rutherford, NJ, USA).

### Surgical procedure and clinical evaluation

All pre and post procedures were carried out as previously described^52^. Briefly, eight New Zealand White rabbits were used for this study. All CE-CI procedures were performed by JSM. The use of rabbits, their care and treatment adhered to the regulation of the ARVO statement for the Use of Animals in Ophthalmic and Vision Research, and all experimental procedures were approved by the Institutional Animal Care and Use Committee of SingHealth, Singapore (Ref:2017/SHS/1292). All surgical procedures and follow-up evaluations were performed under general anesthesia achieved by intramuscular injections of 5 mg/kg xylazine hydrochloride (Troy Laboratories, New South Wales, Australia) and 50 mg/kg ketamine hydrochloride (Parnell Laboratories, New South Wales, Australia), along with topical application of lignocaine hydrochloride 1% (Pfizer Laboratories, New York, USA).

### Lens extraction surgery

The crystalline lenses of rabbits were extracted using a standard phacoemulsification technique as previously described^17^. Mydriasis was achieved by the administration of tropicamide 1% (Alcon Laboratories, Texas, USA) and phenylephrine hydrochloride 2.5% (Alcon Laboratories) eye drops approximately 30 minutes before surgery. A clear corneal incision was made with a 2.8 mm disposable keratome. A 5.0 mm diameter continuous curvilinear capsulotomy of the anterior capsule was created under viscoelastic material (Viscoat; Alcon Laboratories) instilled into the anterior chamber. Hydro-dissection was performed using a 27-gauge cannula. The lens was then aspirated and removed with a standard phacoemulsification procedure using the White Star phacoemulsification system (Abbott Medical Optics, California, USA). Subsequently, the corneal incision was sutured with 10/0 nylon suture and the rabbits were left aphakic with an intact posterior capsule for at least one week before the CE-CI procedures. Cataract extraction surgery of the rabbits were performed by either HSO or FMW.

### Corneal endothelial cell injection procedure

The delivery of CE-CI was carried out as previously described^52^. Briefly, rabbits received an intravenous dose of heparin (500 units in 1.0 ml). To prevent collapse of the anterior chamber (AC) the AC was infused with heparin containing, balanced salt solution (BSS). To remove the rabbit’s endogenous CE, a paracentesis was first created with a diamond knife to accommodate the insertion of a 30-gauge silicone soft tipped cannula (ASICO, Illinois, USA). The rabbit CE was carefully scrapped off, keeping the DM intact in a limbus to limbus direction. Continuous irrigation with BSS ensured endothelial cells did not remain on the surface of the DM. Intracamerally injection of trypan blue was used to aid in the assessment of the DM denudation. The process of scrapping was repeated until the all rabbit’s CE was removed^52^. Subsequently, 0.5 mL of 100 μg/mL carbochol (Miostat, Alcon Laboartories) was injected to achieve intraoperative miosis. Both the paracentesis incision and the anterior chamber maintainer paracentesis sites were secured with 10/0 nylon interrupted sutures. This was followed by a 0.2 mL anti-inflammatory and anti-infective subconjunctival injection of a 1:1 mixture of 4 mg/mL dexamethasone sodium phosphate (Hospira, Melbourne, Australia) and 40 mg/mL gentamicin sulfate (Shin Poong Pharmaceutical, Seoul, Korea). Using a syringe and 30 G cannula, 0.4 ml of aqueous humour was removed to shallow the anterior chamber.

To assess the functionality of HCEnCs following 48 hours hypothermic storage at 4 °C, post-storage recovery of HCEnCs was first achieved by seeding HCEnCs into an organ culture dish (Corning, NY, USA) pre-coated with either FNC coating mixture or Collagen IV. Re-established adherent monolayer of HCEnCs was stabilized and maintained in M5-Endo for at least 2 days before they were dissociated and re-suspended in 100 μl of M5-Endo containing 10 μM ROCK inhibitor. Subsequently, the solution of prepared cells was injected through a separate tunneled track via a 30 G needle. Immediately following CE-CI, rabbits were positioned in a manner that ensured the cornea was in a downward position. Rabbits were maintained in this position for three hours under volatile anesthesia. For this study, treatment group are defined as rabbits that received a single injection cultured P1 or P2 HCEnCs that were subjected to hypothermic storage for at least 48 hours (n = 4). Control group consist of rabbits that had their CE removed by scraping (n = 4), also keeping the DM intact, but without injection of any cells.

### Post-transplantation care

All rabbits received a post-operative regime as previously described^52^. Briefly, topical prednisolone acetate 1% (Allergan Inc, New Jersey, USA) and topical antibiotic tobramycin 1% (Alcon Laboratories) was applied four times a day. An intramuscular injection of 1 mL/kg dexamethasone sodium phosphate (Norbrook Laboratories, Northern Ireland, UK) was administered once daily. This medication regime was maintained for at least 14 days until the rabbits were sacrificed. All corneal imaging and measurements of intra-ocular pressure (IOP) were performed prior to transplantation, as well as at day 4 and 1, and 2 weeks after surgical procedures. Slit lamp photographs were taken with a Zoom Slit Lamp NS-2D (Righton, Tokyo, Japan) and corneal cross-sectional scans and measurements of corneal thickness were performed using an anterior segment optical coherence tomography system (AS-OCT; Optovue, California, USA). Three measurements were taken for the assessment of central corneal thickness (CCT): at the corneal center (0.0 mm), and at 1 mm either side of the center (+1.0 mm, and −1.0 mm), and the mean value reported. Measurements of IOP were measured using a calibrated tonometer (Tono-pen Avia Vet, Reichert Ophthalmic Instruments, New York, USA).

### Statistical analysis

All data is presented as mean ± standard deviation. One-way analysis of variance with repeated measures (ANOVA) with Tukey’s HSD post-hoc test was used to compare groups. Dunnett’s test was used to compare viability levels for HCEnCs in suspension storage with pre-storage viability as the control. Independent-sample *t*-test was used to compare corneal thickness of rabbits receiving CE-CI and rabbits without CE-CI. Significance was set at P ≤ 0.05. All calculations were performed with R software package (R version 3.4.2, R Foundation for Statistical Computing, Vienna, Austria) and Excel software (Microsoft, Redmond, WA, USA).

## Supplementary information

Supplementary Information.
