# Supplementary material for: Optimisation of Storage and Transportation Conditions of Cultured Corneal Endothelial Cells for Cell Replacement Therapy
Source: Sci Rep. 2020 Feb 3;10:1681. doi: 10.1038/s41598-020-58700-5 (PMC6997453; doi:10.1038/s41598-020-58700-5)
Supplement: Supplementary file 1 — Supplementary Information. [file 41598_2020_58700_MOESM1_ESM.docx]

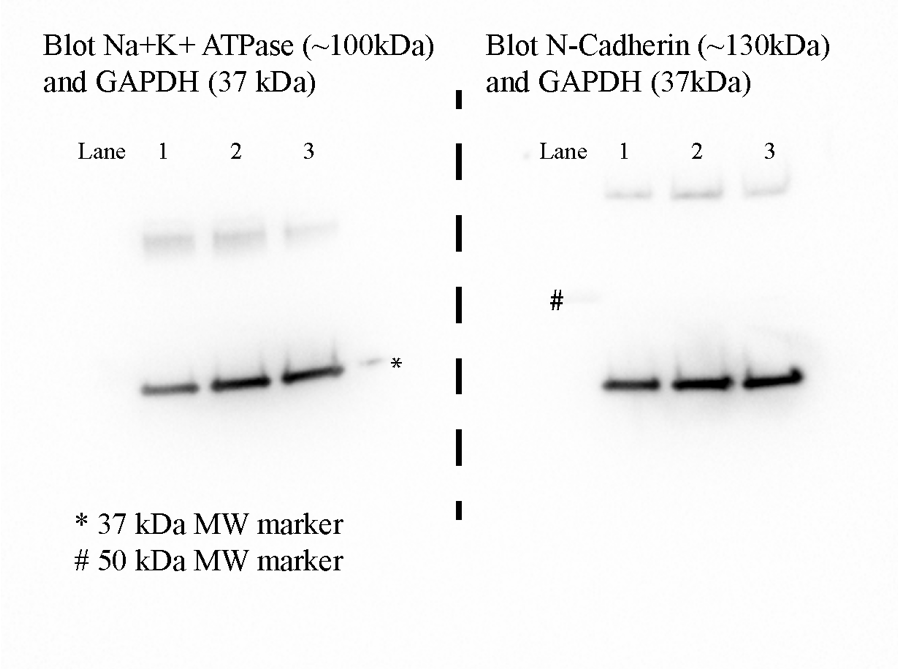


Supplementary figure 1.

Relating to figure 3D. Human corneal cell lysates were subjected to SDS-PAGE in duplicate. Proteins were transferred to PVDF membrane and cut into two halves (dotted line). Membranes were serially probed with antibodies to the indicated proteins. Membranes were visualised by chemiluminescence and imaged on a ChemiDoc™ MP imaging system with image lab software (Bio-Rad). Image exposure time was automatically calculated by imaging software.
